# Biodegradable NR Latex Films with Lignocellulosic and Collagen Hydrolysate Fillers

**DOI:** 10.3390/ma18153711

**Published:** 2025-08-07

**Authors:** Magdalena Kmiotek, Mirosława Prochoń, Elżbieta Sąsiadek-Andrzejczak

**Affiliations:** 1 Centre of Papermaking and Printing, Lodz University of Technology, Wolczanska 221 Street, 93-005 Lodz, Poland; 2 Institute of Polymer and Dye Technology, Faculty of Chemistry, Lodz University of Technology, Stefanowskiego 16, 90-537 Lodz, Poland; miroslawa.prochon@p.lodz.pl; 3 Department of Mechanical Engineering, Informatics and Chemistry of Polymer Materials, Faculty of Material Technologies and Textile Design, Lodz University of Technology, Zeromskiego 116 Street, 90-543 Lodz, Poland; elzbieta.sasiadek@p.lodz.pl

**Keywords:** lignocellulose filler, collagen hydrolysate, natural rubber latex, biodegradation ability, mechanical properties, morphology

## Abstract

The objective of this study was to investigate the influence of the lignocellulose filler originating in wood and non-wood raw materials, alone or together with collagen hydrolysate, on the properties and biodegradation ability of natural rubber latex. The different hydrophobicity of the polymer matrix and natural filler makes it difficult to obtain a homogenous structure of the composite. However, the easy biodegradation of the natural filler is a sufficient reason to seek a compromise between its useful properties and the environmental safety of the material. The composites were filled with lignocellulose filler: pine, spruce, and birch wood flour or willow, raspberry, and mallow non-wood flour. Collagen hydrolysate was used as a substitute for lignocellulosic filler, together or alone. The mechanical properties of the composites, their hardness, and equilibrium swelling were studied. In order to determine the morphology and interactions between filler and latex, scanning electron microscopy together with infrared spectroscopy were engaged. The results revealed that after the incorporation of 4 phr of the filler, the increase in mechanical strength was observed even despite the lack of compatibility between the filler and polymer matrix. The lignocellulose filler is a promising agent because its biodegradability contributes to the overall environmental safety of the polymer material.

## 1. Introduction

Natural rubber latex (NRL) is a natural product obtained by collecting the milky secretion of Pará rubber plants (*Hevea brasiliensis*), formed as a result of mechanical injuries of rubber tree trunks and acting as a wound healing agent. Latex is a colloidal mixture composed of a water-based serum phase and the solid rubber phase [1]. *Hevea* latex consists of about 25–35% (*wt*/*wt*) poly(*cis*-1,4-isoprene), 1–1.8% (*wt*/*wt*) protein, 1–2% (*wt*/*wt*) carbohydrates, 0.4–1.1% (*wt*/*wt*) neutral lipids, 0.5–0.6% (*wt*/*wt*) polar lipids, 0.4–0.6% (*wt*/*wt*) inorganic components, 0.4% (*wt*/*wt*) amino acids, and 50–70% (*wt*/*wt*) water [2,3]. After coagulation of latex, nonlinked but partially vulcanizable polymer chains of 10^6^ Da molecular mass are obtained, presenting elastic properties [2,4].

NRL, due to its unique properties, such as high elasticity, strength and tack, rolling resistance, hydrophobicity, and low gas permeability, is applied in many industries, e.g., electronics, automotive, sports, household, and medical applications (gloves, condoms, catheters, etc.) [1,5].

Since the beginning of the 2000s, rubber production has seen significant growth. According to data from the Food and Agriculture Organization (FAO), global production of natural rubber increased by 109 percent and its harvested area by 72 percent between 2000 and 2020 [6,7]. Global production of natural rubber latex reached 13.9 million tons in 2021 [8]. The production of NRL is concentrated in South and South-East Asia, with Thailand and Indonesia as the main producers, constituting ca. 60% of worldwide production. The main importers of NRL are Malaysia and China [9].

The huge production volume is accompanied by a huge amount of natural rubber waste. In particular, in 2020, the year of the pandemic, 65 billion gloves were consumed monthly worldwide [10,11]. The increased use of personal protective equipment (including latex gloves) has led to an uncontrolled and unmanageable amount of waste, posing a risk to human health and ecosystems when directly discarded in the environment [12,13].

The presence of poly(cis-1,4-isoprene), which is its main constituent, is the main reason for the relative resistance of NRL to microbial decomposition in the environment in comparison to many other natural polymers [14,15]. Not only polymer chains but also the composition of NRL hinder the biodegradation process, leading to the accumulation of rubber waste in landfills and the environment [16,17,18]. For example, to prevent degradation and coagulation of NRL occurring spontaneously in freshly collected latex, stabilizing agents are applied. The most commonly used long-term conservator is ammonia [19,20], which in a concentration above 0.35% by mass acts as a very effective bactericide [21].

Since it is difficult to cleave internal linkages between polymer chains in natural rubber latex (NRL) with the use of microorganisms, not only due to the structure and configuration of NRL but also the composition and effect of different curing agents, the promise of NRL waste management lies in the introduction of a biodegradable filler.

Lignocellulosic fillers are attracting considerable attention as alternative reinforcements in rubber composites due to their renewability, availability, high mechanical properties, low density, and low cost [22,23,24]. Bio-fillers, e.g., chitosan and corn-based fillers, can also improve the biodegradability of NRL composites [25,26]. Besides their biodegradability, NRL products offer a sustainable approach to improving environmental compatibility.

Lignocellulosic fillers, generally classified as non-reinforcing fillers for rubber composites due to the poor compatibility between hydrophobic rubber molecules and hydrophilic fillers, are subjected to physical or chemical modifications aiming to improve their interactions [18,19,20,21]. Nevertheless, these modifications require additional costs and can also decrease the biodegradability of natural fillers [16].

In this research, raw lignocellulosic fillers and collagen hydrolysate were studied as biodegradability-introducing agents for natural rubber latex composites. The aim of this study was to apply the smallest possible amount of filler, just enough to ensure biodegradability, and not too much to avoid a mechanical strength decrease. The application of naturally available fillers like wood and non-wood flours and collagen hydrolysate originating in waste collagen complies with the circular economy, oriented towards renewable resources and waste reduction.

According to our knowledge, such a comprehensive selection of versatile raw materials and their combination with an animal-derived hydrolysis product have not yet been studied in the literature.

## 2. Materials and Methods

Prevulcanized natural rubber latex Revultex LAN was purchased from Torimex (Torimex Group Sp. z o.o., Konstantynów Łódzki, Poland; medium modulus natural latex, characterized by low ammonia content, i.e., 0.38%, pH 10.5; solid content 60.5%, Ford Cup 3 viscosity at 25 °C 32.5 s, mechanical stability min. 800 s).

Wood (pine (*Pinus sylvestris*) P, spruce (*Picea abies*) S, or birch (*Betula pendula*) B) and non-wood (willow (*Salix cinerea*) W, raspberry stalk (*Rubus idaeus ‘Polana’)* RSS, mallow (*Sida hermaphrodita*) M) raw materials were kindly supplied by a local sawmill (DrewnoHurt, Lodz, Poland) and farmers, who would like to stay anonymous. *Virginia fanpetals* (mallow) originated in the Felin experimental plantation of the University of Life Sciences in Lublin and was kindly supplied by Roman Molas.

Lignocellulosic flours were obtained by grinding the selected raw material in the Körner laboratory mill (Maschinenfabrik Körner HSC GmbH, Barchfeld, Germany) and subsequent sieving to obtain a grain size between 0.1 and 0.4 mm.

To determine the chemical composition of lignocellulosic fillers, the content of moisture was set in accordance with the ISO 287:2017 standard [27], followed by the resolution of extractives (in acetone) (Tappi T280 pm-99) [28], substances soluble in 1% NaOH (Tappi T212 om-12) [29], cellulose with Seifert’s method [30], acid-insoluble lignin, and ash contents according to Tappi T222 om-15 and T211 om-12 [31], respectively. The presented results are arithmetic averages from the measurements, of which there were at least two repetitions. The results do not differ by more than 0.2%.

Collagen hydrolysate was produced according to the procedure given in the Mirosława Prochoń patent [32]. To produce collagen hydrolysate, chromate-treated skin was used, which was fleshing waste. The material was ground in a Retsch MM400 grinder (RETSCH GmbH, Haan, Germany) at a frequency of 60 shakes per second for 1 min. Hydrolysis was carried out in two stages. In the first stage, alkaline hydrolysis in 0.25 M NaOH (pure for analysis, Eurochem BGD Sp. z o.o., Tarnów, Poland) at 85 °C for 2.5 h was performed. After this time, the pH was adjusted to 9 using concentrated sulfuric acid (min. 77%, PCC Group, Brzeg Dolny, Poland). In the second step, enzymatic hydrolysis was carried out with the NUE12 MP enzyme (3,2% mass, proteolytic enzyme, Novozymes A/S, Bgsværd, Denmark) at 50 °C for 3 h. Collagen hydrolysate was dried in a laboratory dryer (thermal chamber, BINDER GmbH, Tuttlingen, Germany) for 48 h.

Surface free energy (SFE) of lignocellulosic fillers and collagen hydrolysate was calculated on the basis of contact angle measurements in a tensiometer Kruss K100 MKII (Kruss GmBH, Hamburg, Germany). The contact angle and the surface free energy were calculated based on the sum of acid-base polar (*γp*) and Lifshitz–van der Waals non-polar (*γd*) interactions. Deionized water acted as the polar and non-polar (dispersive) probe (since 63.7% of the total surface tension of water is polar), and diiodomethane was considered as a non-polar-only (*γpl* = 0) dispersive liquid.

NRL films were obtained by mechanically stirring (IKA RW16 Basic, IKA-Werke GmbH & Co. KG, Staufen, Germany) the latex with lignocellulosic fillers in a 4 phr amount, alone or totally/up to half replaced with collagen hydrolysate, and poured into a Petri dish. Then, the mixes were left alone for 24 h at room temperature in order to cure. The composition of the films is given in Table 1.

Equilibrium swelling in toluene was conducted according to the procedure known and applied for years in the Institute of Polymer and Dye Technology of Lodz University of Technology [33]. On its basis, the volume fraction of cured polymer in the swollen sample was calculated, giving the information on its curing degree.

The mechanical strength at break, elongation at break, and modulus were tested with the Zwick Universal Mechanical Testing Machine with the use of dumbbell-shaped specimens of 3 mm width, according to the PN-ISO 37:1998 standard [34].

The Shore A hardness was measured with a Zwick/Roel electronic hardness tester working with a pressure force of 12.5 N in accordance with the PN-71/C-04238 standard [35].

The Nicolet 6700 spectrophotometer (Thermo Scientific, Waltham, MA, USA) was utilized to analyze the structure and functional group interactions within the macromolecular segments of the gel materials. Measurements were performed in the FTIR wavelength range of 4000–400 cm^−1^ with a resolution of 0.25 cm^−1^.

The cross-section morphology of the latex samples was analyzed with a TESCAN VEGA3–EasyProbe (TESCAN Brno, s.r.o., Brno, Czech Republic) scanning electron microscope (SEM) equipped with VEGATG software (version 4.2.4.0; high-vacuum mode (SE); accelerating voltage 20 kV). The surface morphology of the samples was analyzed with a JSM–IT200 (In-Touch-Scope™, high-vacuum mode (SE); accelerating voltage 10 kV, JEOL Ltd., Tokyo, Japan). Before the measurements, the samples were coated with Au-Pd layers using a Cressington Sputter Coater 108 auto system (Cressington Scientific Instruments Ltd., Watford, UK).

The dispersions of the lignocellulosic filler and/or collagen hydrolysate in the natural rubber latex were investigated using the Nano AFM (Nanosurf, Liestal, Switzerland) atomic force microscope (AFM) in amplitude and the phase mode. The resonant frequency was 190 kHz. The AFM images were interpreted according to the procedure described by Horcas et al. [36].

Biodegradation ability tests were performed under aerobic conditions in garden soil (Pokon, Poznań, Poland) (pH 6.5). The contents of humus (organic humus), nitrogen, phosphorus, potassium, and organic matter in the soil were 5–15%, 0.1–0.3%, 30–60 mg/kg, 50–150 mg/kg, and 30–40%, respectively. The structure of soil was loose, well-drained, and rich in organic matter. The tested material was exposed under laboratory conditions in a climatic chamber (MEMMERT type HPP 108, Memmert GmbH + Co. KG, Schwabach, Germany) at an air humidity of 80% and a temperature of 30 °C. The specimens were cut into 0.2 to 1.7 g mass pieces (weighted on an analytical scale) and placed in laboratory cuvettes filled with soil in a weight ratio of soil to samples reaching 6:1. The samples were kept at 23 ± 2 °C and exposed to saturated humidity obtained with a regular spray application of water. After 30 days of exposition, they were drawn from the cuvettes, cleaned with a brush, dried in the air, and weighted on a laboratory analytical scale. As the biodegradability index (*I_B_*), the relative weight loss was calculated:

(1) $$I_{B}=\;\frac{m_{0}-\;m_{B}}{m_{0}}\;\cdot 100\%$$

where *m*_0_—initial weight of the sample, g;

*m_B_*—weight of the biodegraded sample, g.

To determine the micro-colonization patterns, SEM micrographs were taken according to the procedure given previously.

## 3. Results

The incorporation of wood and non-wood raw materials into rubber latex led to a change in the mechanical properties of the composites. In the case of wood flour’s addition to the NRL, tensile strength at break raised from 9.832 MPa to 11.521 MPa, which stands for a 17% improvement (Table 2). However, the incorporation of willow does not improve the mechanical strength at break, and the worst situation is observed after the addition of raspberry stalks and mallow. In the last case, TS_b_ reached almost half of the initial strength at break of the latex. The poor situation is also observed after the addition of collagen hydrolysate. In this blend, a decrease of almost 70% was detected. However, when collagen hydrolysate was applied together with pine flour, the value reached 7.540 MPa, which stands for more than 75% of the TS_b_ obtained for NR latex alone.

Elongation at break increased for samples containing pine, spruce, birch, willow, raspberry stalks’ sawdust, or collagen hydrolysate. In these cases, the presence of lignocellulosic fillers weakened the interactions between polymers chains, acting similar to plasticizers [37,38], without disturbing the mechanical strength of the NRL. During a mechanical strength test, a crystal phase (stress-induced crystallization) [39,40] in NRL formed, which neutralized the negative influence of plant sawdust on tensile strength at break. Only for NRL MS and NRL CH SS did elongation at break decrease. For these samples, the hardness was the lowest, too (Table 2).

After the addition of wood or non-wood flours and collagen hydrolysate together or alone, the hardness of the samples was changed. In the case of pine, spruce, birch, and willow, an increase, by up to 25%, was observed. On the other hand, after the incorporation of non-wood flours, the decrease was observed. The same was noted for the collagen hydrolysate-containing sample. The combination of collagen hydrolysate and pine flour resulted in a moderate decrease in the hardness in comparison to the initial value. The deterioration of mechanical properties of the NRL composites was connected with the decrease in curing density. The volume fraction of cured NRL decreased by c.a. 15% after the incorporation of natural fillers (Table 2).

The incorporation of organic fillers into NRL led to an increase in water content (Table 2). The moisture content was almost three times higher in the case of the willow sawdust-containing sample than for NRL. However, there was no correlation between moisture content and TS_B_ worsening. For NRL CH and NRL CH SS, the water content was almost the same, but mechanical strength at break was totally different. This indicates that not only the water content but also other factors play a crucial role in the mechanical strength creation of the composites.

The decrease in curing degree and mechanical properties of the wood/non-wood composites was probably connected with the different hydrophobicity degrees of the samples. The natural rubber latex surface free energy (SFE) is relatively high and makes it difficult to wet down certain surfaces. However, the SFE of pine, spruce, and birch sawdust is close to this parameter, known for natural rubber latex and equal to 32.2 mJ/m^2^ [41]. For composites containing these lignocellulosic fillers, mechanical properties were at their best (see Table 2). The lowest TS_B_ was measured for the collagen hydrolysate and willow sawdust-containing samples (CH and WS, respectively), and their surface free energy was as low as 21.9 and 16.1, respectively, which were 10 and 16.1 mJ/m^2^ lower than the SFE for NRL. For NRL RSS, TS_B_ was also lower than for NRL. For these fillers, the difference in SFE was more than 5 mJ/m2 (Table 3). However, the polar component of SFE for NRL (6.5 mJ/m^2^) was definitely lower for the hydrocellulosic fillers (Table 3).

**Figure 1:**
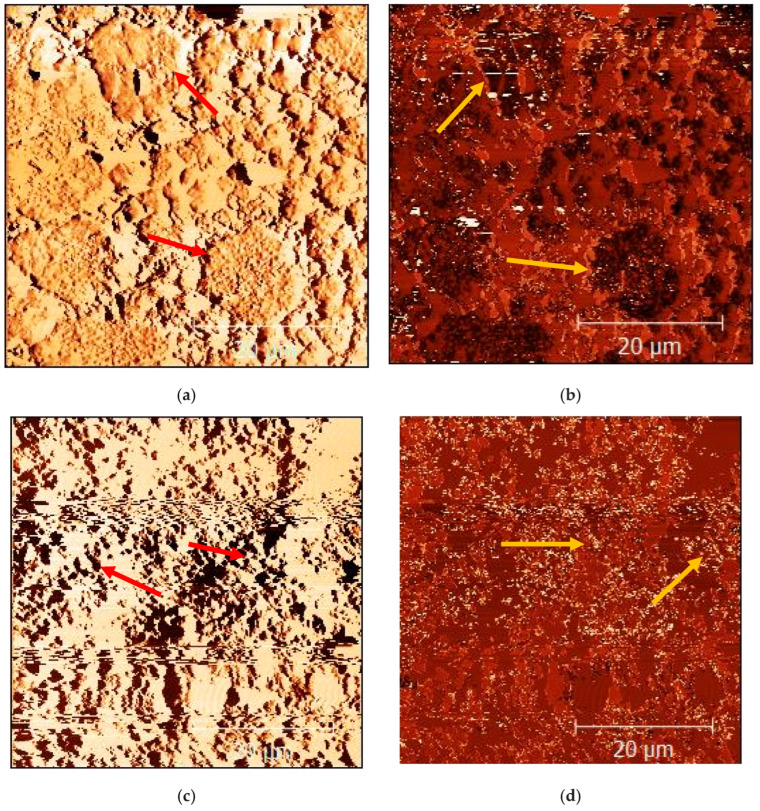
Dispersion of spruce sawdust (**a**,**b**) and collagen hydrolysate (**c**,**d**) in natural rubber latex; (**a**,**c**)—amplitude mode; (**b**,**d**)—phase mode.

Despite poor interfacial interactions between the NRL matrix and applied fillers (Table 3), the wood, non-wood, and collagen hydrolysate fillers disperse relatively well (Figure 1). Both kinds of fillers do not show an excessive tendency to form agglomerates, which is an effect of weak polymer–filler and filler–filler interactions. In addition, the large size of lignocellulose filler makes it difficult to agglomerate in the polymer matrix. It was evidenced that lignocellulosic fillers are definitely larger than collagen hydrolysate parts (see Figure 1b,d).

**Figure 2:**
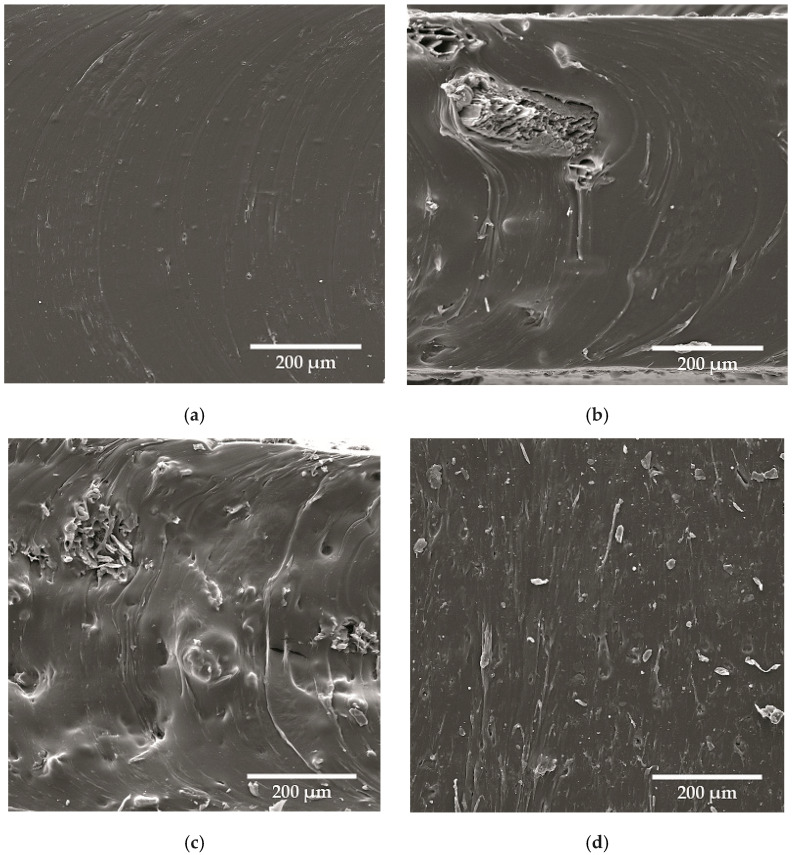
SEM micrographs of natural rubber (**a**) containing sawdust from spruce (**b**), mallow (**c**), and collagen hydrolysate (**d**); magnitude 300×.

The dispersion of the wood/non-wood flours in natural rubber latex was evidenced with SEM micrographs. Despite the poor compatibility resulting from different polarities, the lignocellulosic filler does not form aggregates in the polymer matrix (Figure 2). The particles of the filler, of considerable size, were strongly adhered to the continuous medium of the rubber (Figure 2a). The structure of the sawdust remained unchanged and was penetrated by the natural rubber latex probably before its curing (Figure 2b). The lack of agglomerates and considerable distances between filler parts of the wood/non-wood flours in the latex were probably responsible for the acceptable mechanical properties of the composites. The difference in mechanical strength between mallow and spruce (presenting the lowest and the highest TS_b_ among wood/non-wood flours) can be attributed to the different hardness and compact consistency of the wood sawdust. The mallow flour is very fragile and prone to crushing, which means the shrapnel of the filler causes discontinuities in the composite structure (Figure 2c).

In SEM micrographs of the collagen hydrolysate-containing sample (Figure 2d), small pores in the composite structure were observed, which were probably responsible for the poor mechanical characteristics of the composite containing collagen hydrolysate. One of the key reasons may be the disruption of the polymer cross-linking network. Collagen hydrolysate, as a hydrophilic substance, may interfere with the latex vulcanization process, reducing the cross-linking efficiency (see Table 2) and leading to a weakening of the material structure. Additionally, the presence of micropores resulting from a difference in polarity (see Table 3) and weak adhesion between the phases may promote the initiation and propagation of cracks under the influence of mechanical loading. It is also worth paying attention to the potential plasticization phenomena that may occur as a result of the interaction of hydrophilic collagen with moisture contained in the matrix [42,43]. This may lead to a local decrease in the stiffness of the composite and a further deterioration of its strength properties. After the incorporation of lignocellulosic filler, a increase in TS_b_ was observed, probably due to the interactions between collagen and wood flour, reducing the adverse impact of the former (Figure 2c). The interactions between collagen hydrolysate and wood flour probably covered the surface of the lignocellulosic filler. As a result, poor interactions between the filler parts and polymer matrix occurred. In SEM micrographs, empty spaces between these two fractions could be seen (Figure 2c,d).

**Figure 3:**
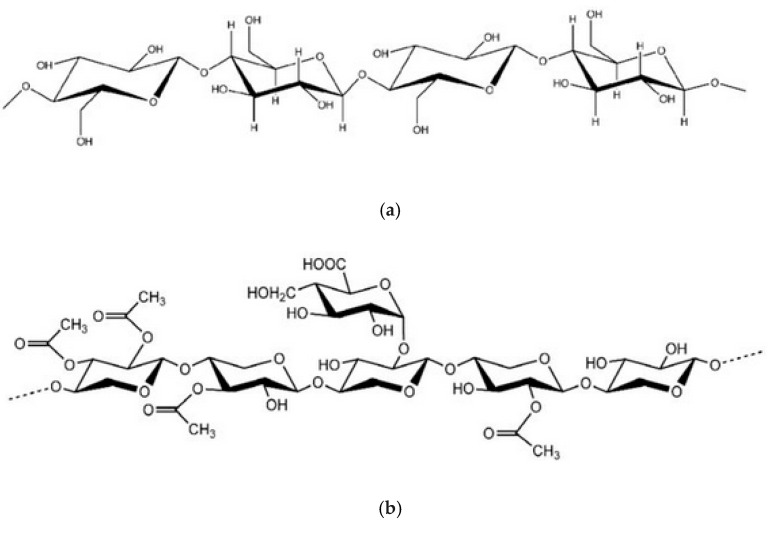

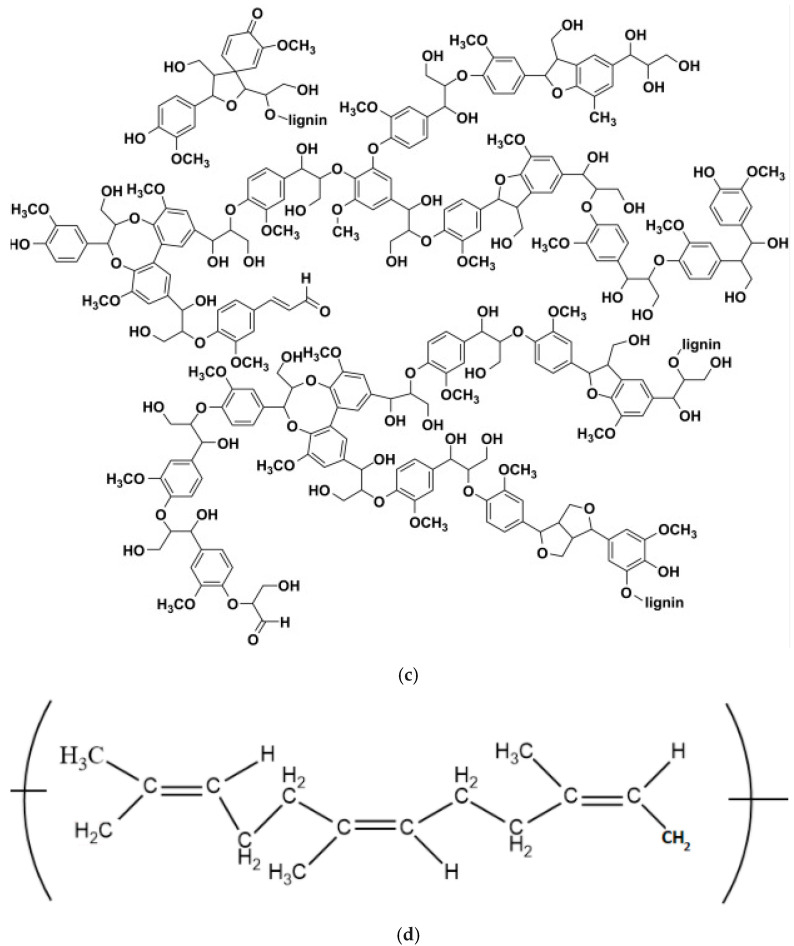
Chemical structure of cellulose (**a**) [44], hemicellulose (**b**) [45], lignin (**c**) [46], and natural rubber (**d**) [47].

The lack of interfacial interactions between lignocellulosic fillers and natural rubber latex follows from the differences in their chemical structure (Figure 3, Table 4).

The fillers contain mainly cellulose, composed of anhydroglucose units joined with beta-1,4-linkages, which exhibit hydrophilic properties originating in hydroxyl groups’ presence in every structural unit (Figure 3a). On the other hand, the presence of hemicellulose in the lignocellulosic filler, significantly high in the case of willow (WS) and raspberry stalks (RSS), imparts hydroxyl groups (from its building units) (Figure 3b). The content of holocellulose exceeds 55%, being the highest for willow sawdust (>75%), enhancing the hydrophilic character of the component. In the filler, the more hydrophobic part is lignin. Its every segment is built by a phenyl propane unit, containing an aromatic ring (Figure 3c). However, every repeating unit contains hydroxyl groups bonded with an aromatic ring or propane unit. The hydrophilic properties of the lignocellulose filler are responsible for poor interactions with the hydrophobic polymer matrix. Natural rubber latex is devoid of hydroxyl groups and any others of dipole character (Figure 3d). So, the only supposed effect is the lack of compatibility and poor interfacial action between the polymer matrix and lignocellulosic fillers.

**Figure 4:**
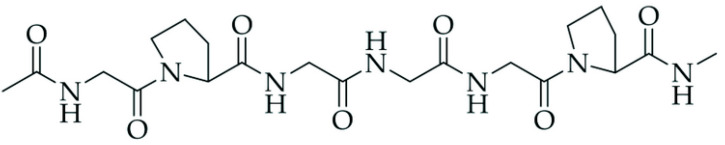
The structure of collagen [48].

After the incorporation of collagen hydrolysate into natural rubber latex, a definite deterioration of mechanical strength was observed, which can be explained by the lack of compatibility between natural rubber chains and collagen hydrolysate particles (Figure 4), resulting from the different chemical structures of the two components.

**Figure 5:**
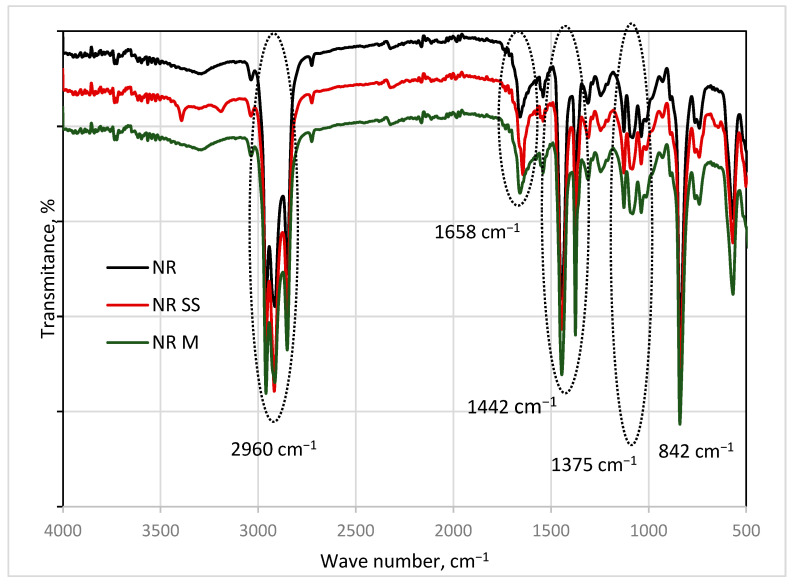
FTIR spectra of natural rubber alone and containing spruce (NR SS) and mallow sawdust (NR M).

From the analysis of FTIR spectra of NRL with and without lignocellulosic fillers, it follows that there are no chemical or physical interactions between the polymer and the filler. In the spectra, absorption bands attributed to natural rubber latex were mainly observed (Figure 5). There is no difference between the NRL spectra and the others containing lignocellulosic fillers. Probably, the characteristic bands of the natural fillers are shielded by strong bands originating in chemical groups present in the latex. However, there are no additional absorption bands and there is no change in the region between 3300 and 3500 cm^−1^, connected with the formation of hydrogen bonds. Only CH, C=C, CH_2_, and CH_3_ groups occur. The functional groups of NRL were detected at the absorption peaks of 2960, 1658, 1442, 1374, and 839 cm^−1^. At 2960 cm^−1^ (CH_3_ group) and 1442 cm^−1^ (C-H), deformation of the bands attributed to cis-polyisoprene was observed [49,50]. The band at 2960 cm^−1^ is connected with stretching vibration of C-H, whereas at 1658 cm^−1^, the C=C diene band was revealed. The characteristic bands of lignocellulosic filler, attributed to -CH_2_ stretching and bending vibrations, can be seen at 2930 cm^−1^ and 1390 cm^−1^. However, the bands corresponding to -CO vibration were not distinguishable. The characteristic bands and groups attributed to them are shown in Table 5.

**Figure 6:**
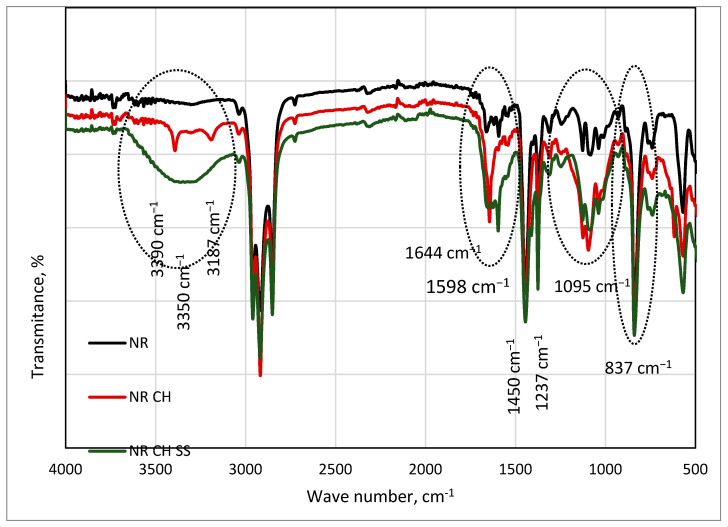
FTIR spectra of natural rubber alone and containing collagen hydrolysate (NR CH) and collagen hydrolysate together with spruce sawdust (NR CH SS).

After the incorporation of the collagen hydrolysate into natural rubber, the FTIR spectra changed (Figure 6). Additionally, before the CH absorption band at 2960 cm^−1^, the absorption bands at 3390 and 3187 cm^−1^ or 3350 cm^−1^ appeared in the spectra of NR CH and NR CHSS, respectively. Novel absorption bands were detected at 1598 cm^−1^, 1237 cm^−1^, and 1095 cm^−1^. The last one was observed in NRL spectra, but after the incorporation of collagen hydrolysate, its intensity and shape changed, suggesting it has a source in different absorption bands. The bands over 3000 cm^−1^ were located in the amide region of collagen (belonging to peptides, containing amide groups). Amide A (3390 cm^−1^) and amide B (3186 cm^−1^) were identified on the basis of the asymmetric stretching of CH_2_ groups and vibration of tension of the NH group in collagen hydrolysate [51,52]. These absorption bands are shielded by a wide absorption band at 3350 cm^−1^ in NR CH SS spectra, connected with the presence of hydrogen bonds between collagen hydrolysate and lignocellulosic filler (namely amide and hydroxyl groups). Three peaks at 2930 cm^−1^, 1390 cm^−1^, and 1030 cm^−1^ correspond to -CH_2_ stretching, -CH2 bending, and C-O stretching, respectively, the presence of which is characteristic of lignocellulosic filler.

At 1644 cm^−1^, an absorption band of C=O was observed in amide I stretching vibration in α-helix chains of collagen, proving its secondary structure. The band at 1598 cm^−1^ is connected with the CN stretching vibration of amide II, whereas the amide III structure was proven by the band at 1237 cm^−1^, corresponding to the stretching vibration of the C-N group and the deformation of the N-H group in amide [38]. The characteristic bands and groups attributed to them are shown in Table 6.

The presence of characteristic bands (Table 6) confirms the secondary structure of collagen on the basis of amide I, amide II, and amide III absorption vibrations’ presence in the FTIR spectra. Almost equal ratios of bands at 1237 cm^−1^ (amide III) and 1450 cm^−1^ (amide II) represent the triple helical structure of collagen [51].

**Figure 7:**
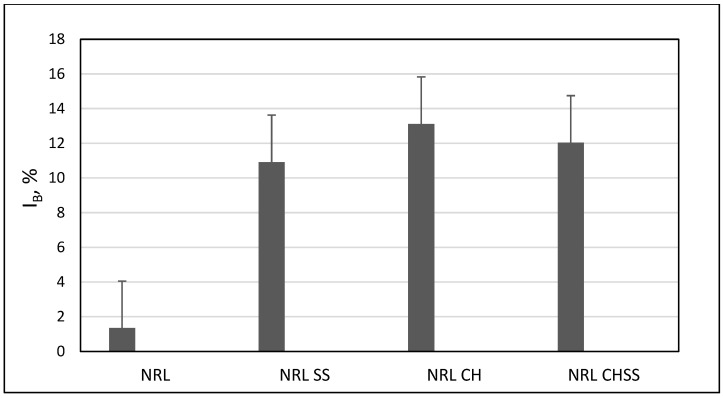
Index of biodegradability of natural rubber alone (NRL) and containing spruce sawdust (NRL SS), collagen hydrolysate (NRL CH), or both fillers together (NRL CHSS).

The biodegradability tests revealed that the incorporation of biodegradable filler, whether collagen hydrolysate or lignocellulosic sawdust, led to an improvement in the biodegradation intensity. The index of biodegradation increased at least 10 times in the case of samples containing flours, collagen hydrolysate, or both of them, in comparison to I_B_ for the NRL sample (Figure 7).

**Figure 8:**
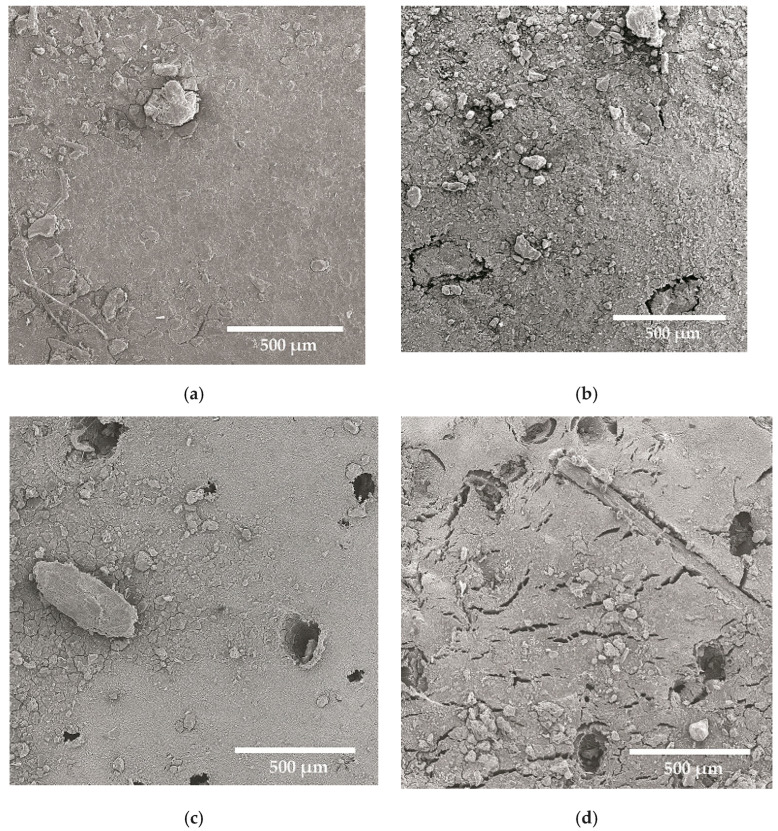
SEM surface micrographs of microorganisms’ colonization of natural rubber (**a**), containing collagen hydrolysate alone (**b**), containing spruce sawdust alone (**c**), and containing both collagen hydrolysate and spruce sawdust (**d**); magnitude 300×.

After biodegradation tests in soil, the surface of NRL changed. While initially smooth (see Figure 2a), after the biodegradation tests, little cracks and filamentous bacteria were spotted (Figure 8a). On the surface of the collagen hydrolysate-containing sample (Figure 2b), much inorganic fouling was observed. No clear individual cells of biofilm could be observed due to extracellular polymeric substances secreted by microorganisms, which made it difficult to distinguish the microbes being deposited, clumped, and glued on the surface. However, the presence of cracks and surface roughness together with a thickness increase indicated biofilm formation. Numerous cracks and holes in the place of lignocellulosic filler occurred in the case of the spruce sawdust-containing sample (Figure 8b). The shape of bacteria was difficult to establish due to their surroundings and secretion of metabolic products. In the vicinity of the filler part on the tested surface, numerous microorganisms could be spotted, indicating the biodegradation mechanism starting with the lignocellulosic filler. The situation was the same with both filler-containing samples. Only holes remained in places with lignocellulosic filler. The biofilm formation was accomplished by sample thickening. The shapes of microorganisms were difficult to define. However, rod-like bacteria together with cylindrical-shaped ones could be seen on the surface of the sample.

**Figure 9:**
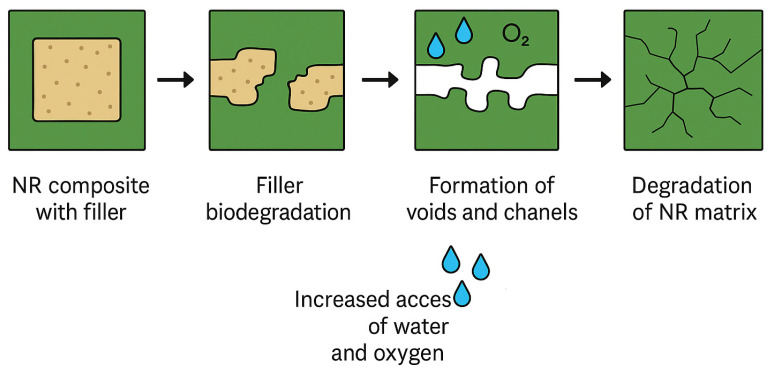
The biodegradation mechanism of NRL containing lignocellulosic and polypeptide fillers.

The mechanism of enhanced biodegradability for natural rubber latex composites reinforced with natural fillers such as lignocellulosic and polypeptide derivatives operates by increasing the surface area for contact of the entire composite with microorganisms and other environmental factors (Figure 9). This mechanism facilitates the access of water and oxygen to the structure’s interior, which can subsequently lead to physical disintegration of the composite, ultimately promoting the decomposition of the NRL matrix. Therefore, the additives used act as degradation initiators at first, and decompose themselves, and the resulting voids accelerate the degradation of the NRL matrix.

## 4. Conclusions

The introduction of natural fillers, such as collagen hydrolysate and lignocellulosic sawdust, is a promising method for improving the biodegradability of rubber while maintaining acceptable functional properties. Despite our expectations, the addition of 4 phr did not deteriorate the mechanical strength at break; in fact, it even improved. Although the hydrophobicity of the filler and rubber matrix are extremely different, the dispersion of the flours and collagen hydrolysate is adequate. A small amount of filler is sufficient to increase the biodegradability of the tested material. Moreover, the results suggest that the appropriate selection of the type and amount of natural additives can enable the optimization of the balance between mechanical strength and biodegradability. It is also possible that the interactions between the composite components can be modified by using additional cross-linking agents or compatibilizers, which could further improve the structural integrity of the material and influence the rate of biodegradation.

In addition, the introduction of organic fillers can contribute to reducing the negative impact of the rubber industry on the environment, offering more ecological alternatives to traditional, synthetic additives. The results obtained suggest that even small additions of organic fillers to natural rubber latex or other rubber materials, especially those containing reinforcing fillers such as carbon black or silica, can lead to a material with improved properties and increased biodegradability. Further studies should focus on the long-term mechanical stability of such composites and their behavior under real operating conditions.

## Figures and Tables

**Table 1:** Composition of the natural rubber latex composites filled with wood/non-wood flours and/or collagen hydrolysate (amounts in g).

	NRL	NRL F	NRL CH	NRL CHF
NR latex	100	100	100	100
Wood/non-wood flour	-	4	-	2
Collagen hydrolysate	-	-	4	2

F can be pine (PS), spruce (SS), birch (BS), willow (WS), mallow (MS), or raspberry stalks’ (RSS) sawdust.

**Table 2:** Mechanical properties and volume fraction of cured polymer for natural rubber latex composites filled with wood/non-wood flours and/or collagen hydrolysate.

	TS_b_, MPa	E_b_, %	°Shore A Hardness	V_r_	Water Content, %
NRL	9.832 ± 1.185	614 ± 2	42.42	0.172	0.69
NRL PS	11.180 ± 1.548	704 ± 55	50.14	0.149	1.54
NRL SS	11.521 ± 0.598	1260 ± 99	53.06	0.152	0.78
NRL BS	11.430 ± 0.851	1620 ± 54	47.78	0.149	1.22
NRL WS	8.941 ± 0.365	1152 ± 151	47.92	0.145	1.90
NRL RSS	7.540 ± 0.871	1209 ± 31	37.88	0.149	1.52
NRL MS	4.453 ± 0.723	607 ± 32	43.00	0.148	1.12
NRL CH	2.459 ± 0.323	1209 ± 32	37.88	0.148	1.55
NRL CH SS	7.540 ± 0.841	570 ± 46	39.30	0.145	1.54

V_r_—volume fraction of cured polymer in toluene.

**Table 3:** Surface free energy (SFE) and its polar (γ^p^) and dispersive (γ^d^) components of wood/non-wood flours and collagen hydrolysate.

	SFE, mJ/m^2^	γ^d^, mJ/m^2^	γ^p^, mJ/m^2^
NR latex	32.2	25.8	6.5
PS	32.8	32.6	0.3
SS	32.0	28.7	3.2
BS	34.1	32.7	1.4
WS	16.1	16.0	0.1
RSS	37.2	32.6	0.1
MS	37.2	33.0	4.2
CH	21.9	14.2	7.7

**Table 4:** Chemical composition (%) of lignocellulosic fillers.

	Cellulose	Lignin	Hemicellulose	Extractives	Mineral Substances
PS	40.02	30.59	17.77	2.98	0.26
SS	38.44	29.53	17.01	0.63	0.18
BS	40.48	19.88	18.92	1.66	0.07
WS	28.31	32.25	47.69	6.95	2.36
RSS	34.71	24.33	22.36	3.14	1.68
MS	40.91	22.47	22.40	1.12	0.99

**Table 5:** Characteristic bands in FTIR spectra of natural rubber latex alone (NRL) and containing spruce (NRL SS) or mallow sawdust (NRL MS).

Band Intensity	Wave Number, cm^−1^	Absorption Group	Assignment
Strong	2960	CH	Stretching vibration
Weak	1658	C=C	Stretching vibration
Strong	1444, 1375	CH_2_	Deformation
Strong	842	CH_3_	Out of plane

**Table 6:** Characteristic bands in FTIR spectra of natural rubber latex alone (NRL) and containing collagen hydrolysate (NRL CN) with spruce sawdust (NRL CHSS).

Band Intensity	Wave Number, cm^−1^	Absorption Group	Assignment
Weak	3390	CH_2_ NH	Stretching vibr. Tension amide A
Weak	3187	CH_2_ NH	Stretching vibr. Tension amide B
Medium	1644	C=O	Stretching vib. amide I
Medium	1598	CN	Stretching vib. amide II
Strong	1450	C=O	Stretching vibrations
Strong	1237	CN	Stretching vib. amide III
Weak	1095	OH	Stretching vib.
Strong	837	CH_3_	Out of plane

## Data Availability

The original contributions presented in this study are included in the article. Further inquiries can be directed to the corresponding author.
